# Blood Biomarkers as a Non-Invasive Method for the Assessment of the State of the Fontan Circulation

**DOI:** 10.3390/jcm14020496

**Published:** 2025-01-14

**Authors:** Andrzej Wittczak, Anna Mazurek-Kula, Maciej Banach, Grzegorz Piotrowski, Agata Bielecka-Dabrowa

**Affiliations:** 1 Department of Preventive Cardiology and Lipidology, Medical University of Lodz, 90-419 Lodz, Poland; 2 Department of Cardiology and Congenital Diseases of Adults, Polish Mother’s Memorial Hospital Research Institute, 93-338 Lodz, Poland; 3 Department of Cardiology, Polish Mother’s Memorial Hospital Research Institute, 93-338 Lodz, Poland; 4 Cardiooncology Department, Medical University of Lodz, 90-419 Lodz, Poland; 5 Cardiology Department, Nicolaus Copernicus Memorial Hospital, 93-513 Lodz, Poland

**Keywords:** Fontan circulation, Fontan operation, blood biomarkers, non-invasive methods, prognosis, therapy personalization

## Abstract

The Fontan operation has become the primary palliative treatment for patients with a functionally univentricular heart. The population of patients with Fontan circulation is constantly growing and aging. As the number of Fontan patients surviving into adulthood increases, there is a clear need for research on how best to follow these patients and manage their complications. Monitoring blood biomarkers is a promising method for the non-invasive assessment of the Fontan circulation. In this article, we provide a comprehensive review of the available evidence on this topic. The following biomarkers were included: natriuretic peptides, red blood cell distribution width (RDW), cystatin C, high-sensitivity C-reactive protein, vitamin D, parathyroid hormone, von Willebrand factor, carbohydrate antigen 125, lipoproteins, hepatocyte growth factor, troponins, ST2 protein, galectin-3, adrenomedullin, endothelin-1, components of the renin–angiotensin–aldosterone system, norepinephrine, interleukin 6, tumor necrosis factor α, and uric acid. We did not find strong enough data to propose evidence-based recommendations. Nevertheless, significantly elevated levels of brain natriuretic peptide (BNP)/N-terminal prohormone of BNP (NT-proBNP) are most likely associated with the failure of the Fontan circulation. The use of the RDW is also promising. Several biomarkers appear to be useful in certain clinical presentations. Certainly, robust longitudinal, preferably multicenter, prospective studies are needed to determine the sensitivity, specificity, evidence-based cut-off values and overall predictive value of different biomarkers in monitoring Fontan physiology.

## 1. Introduction

Introduced in 1968, the Fontan operation has become the primary palliative treatment for patients with a functionally univentricular heart. The operation separates the pulmonary and systemic circulations by creating a venous–pulmonary connection, allowing passive delivery of deoxygenated blood to the lungs while the single functional ventricle maintains systemic circulation [1,2]. The introduction of Fontan palliation has significantly improved the survival of patients with all types of single-ventricle anatomy, including those with underdeveloped or absent right or left ventricles [3]. The Fontan operation was first described by Fontan and Baudet in 1971 for the innovative repair of tricuspid atresia [4]. Since then, the Fontan procedure has undergone several modifications [5]. Today, 50 years after the first clinical application of the original procedure, the modified Fontan operation is one of the most frequently performed procedures in pediatric congenital heart surgery [6]. The worldwide population of patients with Fontan circulation grew to an estimated 50,000 to 70,000 patients in 2018, with 40% of patients >18 years of age [3]. The current estimate of 30-year survival after surgical Fontan completion is about 85% [3]. Consequently, the population of patients with Fontan circulation is constantly growing and aging [7].

As the number of Fontan patients surviving into adulthood increases [7], there is a clear need for research on how best to follow Fontan patients and manage their complications. The authors of the 2020 European Society of Cardiology Guidelines for the management of adult congenital heart disease (ACHD) stated that the care of Fontan patients is one of the major challenges for ACHD practitioners [1]. According to the 2019 Scientific Statement from the American Heart Association (AHA) on Evaluation and Management of the Child and Adult With Fontan Circulation, there is a gap in knowledge in terms of characterizing the state of the Fontan circulation [3]. The authors note that the primary indicators that help physicians assess the quality of the cavopulmonary circulation are invasive measurements of central pulmonary artery pressures and systemic ventricular end-diastolic pressures. However, unless these parameters are significantly abnormal, they have been shown to be poorly correlated with adverse outcomes [3]. Therefore, the authors emphasize the need for a more realistic, dynamic method of characterizing the Fontan circulation that better represents its natural, daily state. They also point out the lack of appropriate metrics for assessing the condition of patients with this unique circulation [3].

Without doubt, there is a continuing need for new methods to assess the status of the Fontan circulation. The monitoring of blood biomarkers is a promising method for the non-invasive assessment of the Fontan circulation, not only for determining current state but also for prognosis and therapy personalization. To date, the issue has only been analyzed in two articles: a 2016 editorial by Schumacher and Goldberg [8] and a short review article published in 2022 by Inai et al. [9]. In this article, we present a comprehensive review of the available evidence on blood biomarkers in patients with Fontan circulation.

## 2. Characterizing the State of the Fontan Circulation—Current Standard

Since the risk of deterioration of the Fontan circulation is substantial, Fontan circulation experts, the authors of the AHA Scientific Statement, proposed a cardiovascular surveillance testing scheme [3]. The following tests were recommended (the suggested frequency depended on the test): electrocardiography (ECG), echocardiography, 24-h Holter ECG monitoring, exercise stress test, serum BNP (brain natriuretic peptide)/NT-proBNP (N-terminal pro-BNP), cardiac magnetic resonance imaging, computed tomography, and cardiac catheterization [3].

Of these methods, invasive cardiac catheterization remains the gold standard for the assessment of the quality of the Fontan circulation. According to the 2020 ESC ACHD guidelines, cardiac catheterization should be performed at a low threshold in cases of exercise deterioration, unexplained edema, new onset arrhythmia, cyanosis, and hemoptysis [1]. This method provides information on hemodynamics (including pulmonary vascular resistance), ventricular and valvular function, the obstruction of the Fontan tunnel, and anomalous vascular connections [1].

However, not only is cardiac catheterization invasive, but its parameters correlate poorly with adverse outcomes unless they are grossly abnormal [3]. Catheterization parameters are also likely to be unreliable and highly variable, even within the same patient, because they are typically measured at a single time point when most patients are fasting, supine, and often under sedation or general anesthesia [3]. In fact, we lack appropriate metrics to characterize the condition of patients with Fontan circulation [3]. Interestingly, the study by Elder et al. showed that cardiologists are marginally able to predict which Fontan patients are at risk for major adverse events over a one-year period [10]. Undoubtedly, there is a need for improved risk stratification model for Fontan patients.

## 3. Fontan Failure—Definition and Diagnosis

Fontan failure can be defined as the physiological impairment that results from the development of chronically elevated central venous pressure (CVP) and low cardiac output [11]. However, it should be noted that the term “Fontan failure” has been used for a wide range of clinical presentations—some authors have used it to describe isolated exercise intolerance, while others have used it to describe late stages of failure, such as symptoms of severe heart failure and major Fontan-specific complications such as protein-losing enteropathy (PLE) and plastic bronchitis (PB) [3,11]. In general, failing Fontan hemodynamics are marked by significant hemodynamic abnormalities, including elevated central venous pressure (CVP), reduced cardiac output (CO), and low oxygen saturation (SaO_2_) [12].

The current view on the complex issue of Fontan failure was systematized in the article by Puyvelde et al. [13]. Fontan failure was defined as symptomatic insufficiency of the total cavopulmonary connection (TCPC) resulting in one or more of the following events: NYHA Function Class III/IV; protein-losing enteropathy (PLE) or plastic bronchitis (PB); Fontan takedown; heart transplantation; or death. The authors categorized the underlying causes of failure into five types: (1) Fontan obstruction; (2) atrioventricular valve regurgitation; (3) systolic ventricular dysfunction; (4) high pulmonary vascular resistance; and (5) restrictive pathophysiology. However, it was emphasized that multiple causes of failure may coexist and interact, making it difficult to determine the specific type of failure for a given clinical presentation [13].

Although cardiac catheterization is recommended in the presence of clinical deterioration in Fontan patients [1], it is important to emphasize that there is no strong evidence of the prognostic value of performing this invasive procedure in stable patients. At the same time, the measurement of selected biomarkers appears to be a promising method for non-invasive assessment. With approximately 10% of patients with an extracardiac Fontan circulation predicted to develop Fontan failure within 15 years [13], there is a clear need to effectively identify patients at risk early and provide personalized care.

## 4. Overview of the Term “Biomarker” and Review Methodology

The term “biomarker” is a blend word of “biological” and “marker” and refers to a broad subcategory of medical signs, that is, objective indications of health status that can be measured accurately and consistently [14]. In the clinical setting, the term “biomarker” is typically used to describe substances whose concentrations are determined in the laboratory, most commonly in serum/plasma, but also in other body fluids or tissues.

An ideal clinical biomarker should have the following characteristics: (1) clinical relevance (there is evidence to support a theoretically sound rationale for its use); (2) acceptable sensitivity and specificity; (3) reliability (defined as the ability to analytically measure the biomarker or changes in the biomarker with acceptable accuracy, precision, robustness, and reproducibility); (4) practicality (preferably noninvasiveness); (5) simplicity (for regular use without requiring advanced equipment or operator expertise, significant time investment, or high measurement costs) [15]. In the case of blood biomarkers, practicality is almost ideal because blood tests are non-invasive and routine. Other characteristics depend on the biomarker, and determining them for a given biomarker is critical in the search for useful tests.

To review the available evidence on blood biomarkers in Fontan circulation, we performed a comprehensive database search on this topic. The MEDLINE database (PubMed) and Web of Science were used. First, the search query “fontan circulation AND biomarker” was used (and all results screened) to create a list of biomarkers studied in the Fontan circulation and to identify the most relevant studies. Then, several searches were performed using the following query scheme “fontan circulation AND <name of the given biomarker>” (e.g., Fontan circulation AND natriuretic peptides). Such searches were performed for all included biomarkers. Additional eligible articles were added by a citation search. As the majority of the database searches were performed in the first half of 2024, the additional search was performed in both databases in December 2024—the search query “fontan circulation” was used and all articles published in 2024 were screened (with the intention of including all the most recent available studies). Inclusion criteria were: (1) article published in a peer-reviewed journal; (2) patients with complete Fontan circulation in the study group (either only Fontan patients or congenital heart disease group but then only if a sub-analysis for Fontan patients was performed); (3) data on included biomarker(s) provided in the article. We excluded articles that were not published in English.

## 5. Blood Biomarkers in the Fontan Circulation—Review of Available Evidence on Currently Used Biomarkers

**Figure 1:**
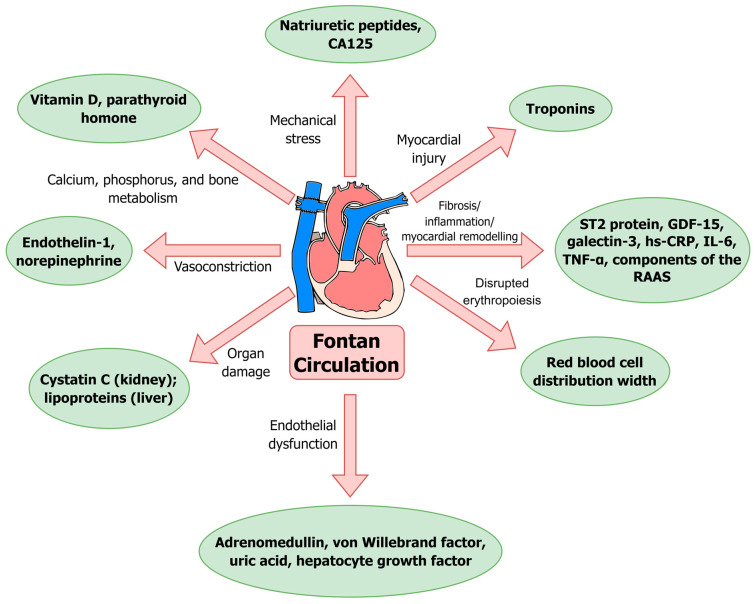
The included biomarkers along with corresponding pathological mechanisms (note that the relationship between a pathological mechanism and a given biomarker is simplified in the figure). Abbreviations: CA125—carbohydrate antigen 125; ST2—suppression of tumorigenicity 2; GDF-15—growth differentiation factor 15; hs-CRP—high-sensitivity C-reactive protein; IL-6—interleukin 6; TNF-α—tumor necrosis factor α; RAAS—renin–angiotensin–aldosterone system.

In Section 5, Section 6 and Section 7, we present a comprehensive review of the available evidence on blood biomarkers in patients with Fontan circulation. The included biomarkers along with the corresponding pathological mechanisms are shown in Figure 1.

### 5.1. Natriuretic Peptides

Natriuretic peptides—atrial natriuretic peptide (ANP) and brain natriuretic peptide (BNP)—are polypeptide hormones produced and secreted by the secretory-contractile phenotype of cardiomyocytes [16]. In clinical practice, the level of the N-terminal prohormone of brain natriuretic peptide (NT-proBNP), which has a longer half-life, is commonly measured [17]. Natriuretic peptides play a role in several physiological functions and are involved in many pathological processes. The concentration of these peptides in the bloodstream has been shown to be an effective diagnostic and prognostic indicator of heart disease, primarily heart failure [18]. In case of ACHD, natriuretic peptides provide crucial prognostic information, but their diagnostic utility for heart failure across diverse cardiac lesions is limited by the cut-off variability, which depends on the underlying defect and the type of repair [1].

As far as biomarkers in the Fontan circulation are concerned, we have the most data on natriuretic peptides.

In 2004, Ohuchi et al. reported that BNP (and norepinephrine) levels distinguished NYHA (New York Heart Association) class II from III/IV in 97 stable Fontan patients; BNP was higher in patients with atriopulmonary connections (APC) compared to those with total cavopulmonary connections (TCPC) [19]. A multivariate analysis showed high BNP and low ejection fraction as key determinants of low NYHA class (*p* < 0.0001).

Inai et al. (2005) found elevated BNP and ANP in 50 Fontan patients (mean age 22.7 ± 3.6 years) [20]. ANP negatively correlated with cardiac index (r = −0.42, *p* = 0.0095). The authors suggested that elevated ANP levels likely indicated increased pulmonary vascular resistance, which is one of the major determinants of cardiac output in the Fontan circulation [20]. Law et al. (2006) observed a BNP elevation in patients with single ventricle (SV) patients with systemic ventricular or left-sided heart failure [21]. Larsson et al. found that BNP/NT-proBNP concentrations were related to the NYHA class but not to ventricular function or exercise capacity in 61 patients with ACHD, 18 of whom had Fontan circulation [22]. Koch et al. (2007) observed normal BNP in 81% of 67 Fontan patients, however, elevated levels were associated with higher morbidity and late mortality [23].

Man et al. compared 35 asymptomatic Fontan patients (mean age 13.7 ± 5.3 years) with 34 controls and found higher plasma BNP levels in the Fontan group [24]. Plasma BNP levels correlated negatively with early (E) and late (A) diastolic inflow velocities, as well as left and right annular myocardial tissue velocities, suggesting an inverse relationship between BNP levels and diastolic function of the systemic ventricle in Fontan patients [24].

Lechner et al. measured NT-proBNP levels in 59 pediatric Fontan patients (median age 8.4 years) and found significantly higher levels in those with congestive HF compared to those without (399 pg/mL vs. 96 pg/mL; *p* < 0.01) [25]. NT-proBNP levels also correlated with HF severity quantified using the New York University Pediatric Heart Failure Index (NYUPHFI) (*p* = 0.001). The authors noted that NT-pro-BNP levels of patients with Fontan circulation without CHF were similar to those of healthy children [25].

Trojnarska et al. found no correlation between BNP and cardiopulmonary exercise test parameters in 10 Fontan patients with systemic right ventricle and concluded a limited diagnostic utility of BNP in such patients [26].

In 2011, Atz et al. reported weak correlations between elevated BNP levels and several indicators of adverse outcomes in 510 Fontan patients aged 6–18 years. The authors did not recommend BNP measurement as an outpatient surveillance tool in asymptomatic Fontan patients [27].

Heck et al. included 124 Fontan patients [49 with atriopulmonary connection(APC)/atrioventricular connection (AVC) and 75 patients with total cavopulmonary connection (TCPC)] and found higher NT-proBNP levels in the APC/AVC group compared to TCPC (*p* < 0.001) [28]. Only in TCPC patients did NT-proBNP correlate with atrioventricular valve regurgitation (r = 0.29, *p* = 0.013) and ventricular dysfunction (r = 0.23, *p* = 0.052).

Kolcz et al. studied 76 TCPC Fontan patients and found a significant correlation between NT-proBNP levels and respiratory equivalent of carbon dioxide at peak exercise (VE/VCO_2peak_) during CPET (cardiopulmonary exercise testing) [r = 0.88, *p* = 0.02] [29]. Since VE/VCO_2peak_ is a sensitive prognostic marker, the authors suggested that NT-proBNP may help to identify high-risk Fontan patients during long-term follow-up.

Burchill et al. studied 106 adult Fontan patients and found elevated BNP (>100 pg/mL) in 33% of patients; elevated BNP was associated with older-style Fontan connections, older age, active arrhythmia, and reduced peak oxygen uptake on CPET (*p* < 0.05 for each) [30]. Elevated serum BNP was an independent predictor of mortality [HR = 1.25 (95% confidence interval [CI] 1.07–1.47) per 50 unit increase in BNP; *p* = 0.006] and of late Fontan failure [HR = 1.11 (95% CI 1.01–1.23 per 50 unit increase in BNP, *p* = 0.04]. The authors emphasized that their study supported the hypothesis that elevated BNP is an independent predictor of Fontan failure and mortality in adult patients [30].

Ohuchi et al. included 197 pediatric and 102 adult Fontan patients and found that serum BNP (per 10 pg/mL) predicted all-cause mortality [hazard ratio (HR) = 1.11 (95% Cl 1.06–1.16), *p* = 0.0002] [31]. Among the examined parameters, BNP was the only independent predictor of all-cause mortality [in the multivariate model: HR = 1.10 (95% CI 1.03–1.18), *p* = 0.0105.

In the study by Miyamoto et al., the authors included 103 ACHD patients, 53 of whom had Fontan circulation [32]. In patients with single ventricle physiology, BNP levels were associated with a higher risk of mortality in univariate analysis [HR = 4.75 (95% CI 1.48–15.27); *p* = 0.009]. In addition, patients with a systemic right ventricle had higher BNP levels than those with a systemic left ventricle (*p*< 0.05). The authors emphasized that the predictors of mortality may vary according to morphological and physiological differences in the ventricles.

Van De Bruaene et al. enrolled 126 ACHD patients (30 with Fontan circulation) and found that BNP > 164 pg/mL was independently associated with the primary outcome (i.e., death, transplantation) [33]. However, BNP was less predictive in Fontan and cyanotic patients, leading the authors to suggest that BNP should perhaps be given less weight in HF decision making in this subgroup.

Baggen et al. found that among the biomarkers tested, NT-proBNP in the upper quartile (>33.3 pmol/L) was most strongly associated with death/heart failure and cardiovascular events in 595 patients with ACHD, including 36 with Fontan circulation [34].

Wolff et al. studied 95 Fontan patients (median age 21.6 years) and found elevated NT-proBNP Z-scores in NYHA class III/IV patients but no correlation with ventricular function or peak exercise capacity [35]. However, peptide Z-scores significantly correlated with right ventricular morphology, follow-up duration after Fontan completion, indexed ventricular mass, and inferior caval vein diameter. The authors concluded that these factors were key to the performance of the Fontan circulation, and therefore elevated NT-proBNP may indicate circulatory failure independent of ventricular function [35].

Nguyen et al. compared biomarker profiles in patients hospitalized for decompensated heart failure, including 54 encounters for 20 Fontan patients and 108 encounters for 108 unique non-Fontan patients [36]. Fontan patients had lower mean admission BNP levels (390.0 ± 978.7 pg/mL vs. 1245.6 ± 1160.7 pg/mL; *p* < 0.0001) and smaller BNP changes from outpatient levels (65.7 ± 185.7 pg/mL vs. 1638.0 ± 1444.7 pg/mL; *p* < 0.0001). The authors concluded that no definitive serologic markers were currently available to identify failing Fontan physiology. Therefore, BNP levels should be used with caution in the evaluation of decompensated Fontan failure [36].

In 2021, van den Bosch et al. studied 133 Fontan patients (median age 13.2 years) using stress cardiac magnetic resonance imaging, CPET, and biomarkers [37]. NT-proBNP was associated with all adverse cardiac events analyzed and remained predictive after adjustment for age, sex, and dominant ventricle (HR 1.89, 95% CI 1.32–2.68; *p* = 0.001). The authors concluded that of all the biomarkers studied, NT-proBNP may have the most significant role in clinical follow-up and risk stratification of patients who have undergone the Fontan procedure [37].

Ghelani et al. studied 82 Fontan patients (median age 18 years) and found that NT-proBNP, along with high-sensitivity troponin T, strongly correlated with ventricular dilation and dysfunction [38]. NT-proBNP > 100 pg/mL showed 82% sensitivity and 45% specificity for detecting reduced ventricular function and 91% sensitivity and 47% specificity for significant ventricular dilation. The authors suggested the use of that NT-proBNP cutoff (>100 pg/mL) as a sensitive marker for identifying patients with significant ventricular dilation or dysfunction [38].

Perrone et al. evaluated the effects of an aerobic exercise training program in 12 adult patients with Fontan circulation (only HLHS) [39]. The authors found a significant reduction in NT-proBNP levels measured before and after the 4-week aerobic training program (96.3 ± 66.7 vs. 62.5 ± 46.1 pg/mL; *p* < 0.01).

Cindik et al. studied 28 Fontan patients (mean age 12.8 ± 4.36 years) and 27 controls and found significantly higher NT-proBNP levels in the Fontan group (*p* = 0.0001) [40]. NT-proBNP levels correlated positively with echocardiographic measures of systolic/diastolic dyssynchrony.

Miranda et al. studied 50 adult Fontan patients and found that NT-proBNP levels were not associated with resting hemodynamics and correlated poorly with exercise Fontan pressures and pulmonary artery wedge pressure (PAWP) [41]. However, NT-proBNP ≥ 300 pg/mL was associated with higher Fontan pressures and PAWP during exercise, suggesting that serum biomarkers may help identify individuals with adverse underlying Fontan hemodynamics.

Finally, Palm et al. studied NT-proBNP levels in 289 children undergoing staged Fontan palliation [42]. The study showed that age-adjusted NT-proBNP (zlog-NT-proBNP) reflected the hemodynamic unloading effect of the three surgical stages on the systemic ventricle in univentricular hearts. The authors theorized that this biomarker could serve as a simple, interpretable tool for outpatient monitoring and therapy optimization [42].

In conclusion, while NT-proBNP measurement is frequently conducted in patients with Fontan circulation, there is limited evidence regarding the interpretation of results within that population. Over the course of Fontan palliation, NT-proBNP levels have been shown to generally decrease [43,44]. The authors of the scientific statement from the American Heart Association on the Evaluation and Management of the Child and Adult With Fontan Circulation recommend measuring serum BNP/NT-proBNP levels once in childhood, every 1–3 years in adolescents, and every 1–2 years in adults [3]. At the same time, the document does not provide any recommendations on how to interpret the results [3]. According to the ESC Guidelines for the management of Adult Congenital Heart Disease, although natriuretic peptides are best studied in ACHD patients, they are least useful in patients with Fontan circulation [1]. It should be noted that there are several techniques for the Fontan procedure [3], and older types of Fontan circulation (e.g., atriopulmonary connection), which involve more atrial tissue in the systemic venous pathway, appear to be associated with higher NT-proBNP levels independent of cardiac status [26,28,45]. Nevertheless, significantly elevated levels of BNP/NT-proBNP are most likely associated with the failure of the Fontan circulation. Certainly, more comprehensive prospective data are needed to advance the clinical use of natriuretic peptides in the management of patients with Fontan circulation. Robust longitudinal studies are needed to determine the sensitivity, specificity, evidence-based cut-off values, and overall predictive value of natriuretic peptides in monitoring Fontan physiology.

### 5.2. Red Blood Cell Distribution Width (RDW)

The red blood cell distribution width (RDW) is an index of variation in the erythrocyte volume. It is calculated automatically in a standard complete blood count test by dividing the standard deviation (SD) of the erythrocyte volume by the mean corpuscular volume (MCV) [46]. The RDW represent a reliable index of anisocytosis and is widely used for the differential diagnosis of micro- and normocytic anemias [47]. Interestingly, there are a number of papers about the RDW as a predictor of morbidity and mortality in health and disease [46]. An increased RDW is associated with various cardiovascular diseases such as acute coronary syndromes or heart failure [48]. Higher anisocytosis independently predicted adverse outcomes in several conditions [48]. In case of ACHD, Martínez-Quintana et al. found that the RDW seemed to be a useful and cheap tool to detect low serum apoferritin levels in hypoxemic patients [49].

There are some data on the RDW in Fontan patients. Tomkiewicz-Pajak et al. studied 32 Fontan patients (mean age 25 ± 4.5 years) and 30 matched healthy controls [50]. Fontan patients showed a higher RDW than controls (14.3 ± 2.4% vs. 12.8 ± 0.5%, *p* < 0.001). Among patients with an elevated RDW (>14.5%), iron levels and oxygen saturation were significantly lower. The RDW was also an independent predictor of oxygen uptake (r = −0.39, *p* = 0.008), suggesting a link between the RDW and exercise tolerance in adult Fontan patients. The authors concluded that the RDW was an indicator of iron deficiency in adult patients with Fontan circulation and that it correlated with lower physical activity [50].

Kojima et al. examined the association between an elevated RDW and heart failure in 38 pediatric Fontan patients undergoing routine cardiac catheterization [51]. The RDW was positively correlated with central venous pressure (CVP) and negatively with venous oxygen saturation (SvO2). Patients with a higher RDW had a lower cardiac index (CIx) than those with a normal RDW (3.3 ± 0.1 vs. 3.8 ± 0.2, *p* = 0.0421). A multivariate analysis confirmed the RDW as an independent predictor of both SvO2 and CVP. The authors suggested that the relationship between an increased RDW and elevated CVP, decreased SvO2, and lower CIx might indicate that the RDW could serve as a prognostic indicator for heart failure in the Fontan circulation [51].

In 2023, Fuentes et al. prospectively enrolled 66 patients with Fontan circulation (mean age 27.4 ± 7.8 years) [52]. Regarding the RDW, the study showed that it was significantly associated with the occurrence of the composite event [odds ratio (OR) = 1.75 (95%CI 1.13–3.1); *p* = 0.013]. The authors also calculated the cut-off points with the optimal sensitivity and specificity for the RDW with a result of ≥14.5%. They concluded that patients with RDW levels of ≥14.5% [along with CA125 levels ≥ 20 U/mL and a Fibrosis-4 score (FIB4) ≥ 0.75] had a very high probability of Fontan circulation failure [52].

In conclusion, there are some data on the RDW in the Fontan circulation, although the amount of evidence is limited. Nevertheless, because the RDW is automatically calculated in a standard complete blood count, clinicians should pay attention to the RDW value. It could be an indicator of iron deficiency, which should be confirmed by appropriate testing and treated if recommended. In addition, RDW values ≥14.5% may be an indicator of worse Fontan circulation. Clearly, further studies of the RDW in the Fontan population are needed to provide evidence-based recommendations.

### 5.3. Cystatin C

Cystatin C is a low-molecular-weight protein (13 kilodaltons) that is produced constantly by all nucleated cells, freely filtered at the glomerulus, and metabolized in the proximal tubule [53]. The measured cystatin C concentration can be used to estimate the glomerular filtration rate (GFR), analogous to the widely used creatinine measurement. However, cystatin C levels are thought to be influenced by fewer factors than creatinine levels [53]. In addition to being a marker of renal function, cystatin C appears to be an independent risk marker for cardiovascular outcomes [54,55].

Cystatin C was also studied in the Fontan circulation. In 2017, Opotowsky et al. conducted a study in which the authors researched markers of kidney dysfunction in 70 adult patients with Fontan circulation [56]. The study showed that cystatin C glomerular filtration rate (GFR) was significantly lower in the Fontan group compared with healthy controls (114.2 ± 22.8 vs. 136.3 ± 12.8 mL/min/1.73 m^2^; *p* < 0.0001). Higher cystatin C GFR was associated with a lower risk of incident events (HR per 1SD = 0.66, 95% CI 0.48–0.90); there was no apparent relationship between creatinine GFR and risk of incident events. The authors concluded that in Fontan patients, cystatin C estimates of glomerular filtration rate were associated with adverse outcomes (while creatinine-based estimates are not) [56].

In 2019, Opotowsky et al. evaluated 911 adults with CHD, including 131 with Fontan circulation, to compare the prognostic value of creatinine- and cystatin C-based eGFR (estimated glomerular filtration rate) equations [57]. In Fontan patients, the creatinine-based eGFR (calculated using the Chronic Kidney Disease-Epidemiology Collaboration, CKD-EPI formula) was, on average, 10.3 ± 19.3 mL/min/1.73 m^2^ higher than the cystatin C-based eGFR (with 95% limits of agreement −28.2 to +48.8 mL/min/1.73 m^2^). A higher cystatin C-based eGFR was associated with a lower risk of adverse outcomes (HR = 0.85; 95% CI 0.76–0.95; *p* = 0.005), whereas the creatinine-based eGFR showed no significant association. The authors concluded that their findings, together with existing evidence, supported the use of cystatin C to improve the estimation of the GFR in adults with Fontan circulation, especially when it was expected to influence important clinical decisions [57].

In the study by Chemello et al., the authors analyzed 43 patients with the Fontan circulation, of which 23 patients were diagnosed with Fontan-associated liver disease (FALD) [58]. In patients with FALD, cystatin C levels were significantly increased in comparison to patients without FALD (*p* < 0.05).

In the study by Saraf et al., the authors analyzed biomarker expression in 44 patients with Fontan circulation and 32 healthy controls [59]. In comparison to age-, gender-, and race-matched controls, Fontan patients had significantly elevated levels of cystatin C [438 (IQR 404–481) pg/mL vs. 370 (IQR 322–426) pg/mL; *p* < 0.0001). The authors noted that although cystatin C levels were elevated in Fontan patients, creatinine levels remained within the reference range [59].

Study by Katz et al., which included 100 patients with Fontan circulation, confirmed that the eGFR may be overestimated by creatinine in Fontan and using the cystatin C eGFR was preferable [60].

In conclusion, measurement of cystatin C in Fontan patients appears to be a valuable tool for assessing renal function in this population. This can be explained by the fact that creatinine-based GFR estimation methods rely on a predictable relationship between serum creatinine and GFR. Since creatinine is produced by muscle, creatinine-based eGFR equations are inaccurate if actual muscle mass differs from expected population-based norms [57]. At the same time, the cystatin C eGFR is considered to be independent of muscle mass [57,61]. This feature is extremely valuable in the case of Fontan circulation because these patients have lower lean muscle mass and muscle strength than matched healthy controls [3,57]. Therefore, creatinine-based methods are expected to overestimate the GFR in that population. Nevertheless, according to the Scientific Statement From the American Heart Association, further studies are needed to assess the accuracy and prognostic significance of different eGFRs in the Fontan population [3].

### 5.4. High-Sensitivity C-Reactive Protein (hs-CRP)

C-reactive protein (CRP), an acute-phase reactant, is a member of the pentraxin family and plays a crucial role in innate immunity [62]. It is primarily produced by hepatic cells in response to elevated levels of proinflammatory cytokines [62]. In the clinical setting, CRP measurement is primarily used to aid in the diagnosis and monitoring of infections. The high-sensitivity C-reactive protein (hs-CRP) test is a biochemical assay that provides a highly sensitive measurement of CRP levels. Such a test measures even low levels of inflammation and is considered a biomarker for the risk of heart disease and stroke, among other conditions [62,63]. hs-CRP was also studied in patients with Fontan circulation.

Miyamoto et al. conducted a study (mentioned above), in which the authors included 103 ACHD patients (median age 28 years), of which 53 had the Fontan circulation [32]. The study showed that elevated levels of hs-CRP were significantly associated with the risk of mortality in a univariate analysis in patients with a systemic morphologically left ventricle [HR = 3.51 (95% CI: 1.61–7.67); *p* = 0.002].

In 2018, Opotowsky et al. aimed to assess the association of hs-CRP levels with clinical characteristics and adverse outcomes in 707 ACHD patients, of which 117 had the Fontan circulation [64] (all from the Boston ACHD Biobank [65]). The study found that disease complexity/severity was not linked to elevated hs-CRP levels. Among 499 patients who underwent cardiopulmonary exercise testing, higher hs-CRP levels correlated with lower peak oxygen consumption. Patients with hs-CRP in the highest quartile (≥2.98 mg/L) faced higher risks for combined adverse outcomes (HR = 3.26, 95% CI 2.25–4.70, *p* < 0.0001) and all-cause mortality (HR = 8.04, 95% CI 3.56–18.17, *p* < 0.0001). In the subgroup analysis of Fontan patients, the highest quartile of hs-CRP was similarly associated with an increased risk of the combined outcome (death or non-elective cardiovascular hospitalization) [HR = 3.23, 95% CI 1.69–6.18, *p* = 0.0039]. The authors concluded that elevated hs-CRP levels, an indicator of chronic inflammation, were associated with adverse clinical outcomes in patients with ACHD and that their results suggested that the measurement of hs-CRP may help assess the risk of adverse events in this population [64].

Hauser et al. investigated the effect of meal ingestion on biomarker levels in 15 Fontan patients and 15 controls, also measuring fasting hs-CRP for exploratory purposes [66]. hs-CRP levels were significantly higher in Fontan patients compared to controls [16.5 (IQR: 6.8, 22.7) nmol/L vs. 5.9 (IQR: 2.1, 11.0) nmol/L, respectively; *p* = 0.034].

Finally, in the recent study by Ohuchi et al., the authors enrolled 155 Fontan patients and 44 controls and evaluated gut dysbiosis and selected biomarkers. Regarding CRP, the study showed that high ln-CRP (natural log-transformed CRP) levels predicted a high risk of HF hospitalization [HR 1.64 (95% CI, 1.17–2.26); *p* = 0.004], and patients with high ln-CRP levels ≥ 4.25 (0.07 mg/dL) had a 3.68 times higher risk of HF hospitalization (*p* = 0.002) [67]. 

In conclusion, although the prognostic use of hs-CRP measurement in Fontan patients seems promising, we need more high-quality data to assess the diagnostic/prognostic significance of the test results.

### 5.5. Vitamin D

Vitamin D is a fat-soluble vitamin essential for regulating calcium levels and maintaining bone health [68]. It can be synthesized in the skin from 7-dehydrocholesterol or ingested from food, then it must be activated to 25-hydroxyvitamin D (25-OH-vitamin D) and finally to its active form, 1,25-dihydroxyvitamin D [1,25(OH)_2_D] [69]. In addition to the well-established role of vitamin D in musculoskeletal health, epidemiologic evidence has shown consistent associations of low vitamin D status with increased risk of a variety of common conditions, including cardiovascular, malignant, infectious, metabolic, and autoimmune diseases [70]. In the systematic review by Hazique et al., insufficient vitamin D levels were linked to a higher risk of hospitalizations, death, and subpar clinical results in patients with heart failure [71].

Vitamin D has also been studied in the Fontan circulation, primarily in relation to the musculoskeletal system, which is critical to the long-term health of children and adults after a Fontan operation [3]. In 2014, Avitabile et al. evaluated the body composition of 50 Fontan patients (median age 11.5 years) [72]. Vitamin D deficiency (<20 ng/mL) was found in 12 patients; these patients had lower leg lean mass Z-scores compared to those with normal vitamin D levels (*p* = 0.01). In the subsequent study from the same center, the authors examined bone density in 43 Fontan patients and found that 26% of them were vitamin D-deficient; interestingly, bone and muscle deficits were not associated with vitamin D levels [73]. In 2016, Holler et al. published an article in which the authors retrospectively analyzed the data of 28 Fontan patients (mean age 8.1 ± 5.3 years) who had been screened for vitamin D deficiency [74]. The study showed that the mean serum 25-hydroxyvitamin D level was 14.1 ± 10.4 ng/mL; 70.3% of patients had a vitamin D level below 20 ng/mL. Only skin type was associated with vitamin D deficiency (white patients had higher vitamin D levels). The authors found a significant relationship between serum 25-hydroxyvitamin D and calcium levels (r = 0.434, *p* = 0.044).

Mancilla et al. published an abstract in the *Circulation* journal, in which the authors performed cross-sectional evaluation of 210 Fontan patients (25 patients had current or a history of protein-losing enteropathy—PLE) [75]. In patients without PLE, the mean level of vitamin D was 31.7 ± 11.7 ng/mL. Furthermore, 13% of all patients had low levels of vitamin D (<20 ng/mL).

Diab et al. measured bone mineral density in 64 Fontan patients [76]. Vitamin D levels were insufficient or deficient in 28.5% of patients, and it was deficient in 19.0%. The authors observed an age-related decline in vitamin D levels; they were not consistently associated with bone mineral densities.

D’Ambrosio et al. also measured bone mineral density in 28 Fontan patients [77]. They found 50% of included patients had vitamin D levels below 69.2 nmol/L (the mean vitamin D level in a reference Australian population). In a post hoc correlation analysis, significant inverse correlations between vitamin D and corrected calcium (r = −0.46, *p* = 0.02) and parathyroid hormone (r = −0.53, *p* = 0.01) were found. The authors emphasized that the treatment of vitamin D deficiency, if present, should be standard practice in Fontan patients to improve bone health and reduce the risk of fragility fractures [77].

Weinreb et al. studied exercise performance in 265 Fontan patients, finding 81% had sufficient vitamin D levels (>20 ng/mL) [78]. “High performers” (predicted peak oxygen consumption [VO_2_] ≥80%) had a higher rate of vitamin D sufficiency than others (92% vs. 69%, *p* = 0.001). Vitamin D sufficiency positively impacted peak VO_2_ in both single-variable (effect size = 4.48, *p* < 0.001) and multivariate analyses (effect size = 3.00, *p* = 0.02). The authors hypothesized that vitamin D sufficiency may be protective against sarcopenia in patients with Fontan circulation [78]. Finally, Hansson et al. evaluated vitamin D levels, body composition, liver biomarkers, and leg pain in 44 Fontan children (mean age 12.3 years) and 38 controls [79]. Mean vitamin D intake (9.9 µg/day) met recommendations, and mean serum 25-OH vitamin D was 56 nmol/L, above sufficiency (≥50 nmol/L). However, 42% of Fontan patients had insufficient levels (<50 nmol/L). No association was found between vitamin D levels and bone density, lean/fat mass, or leg pain. The authors noted the high prevalence of low vitamin D despite adequate intake [79].

In conclusion, there are no data on the usefulness of vitamin D as a diagnostic/prognostic biomarker in the Fontan circulation. However, the available studies show that vitamin D deficiency is common in this population, even when vitamin D intake is in accordance with recommendations [79]. At present, it is not known whether there is something unique about the Fontan circulation that affects the absorption of vitamin D. It has been hypothesized that changes in the intestinal circulation may affect vitamin absorption and that differences in renal perfusion may affect vitamin D metabolism in the kidneys [3]. According to the American Heart Association’s scientific statement, understanding how the Fontan operation affects vitamin D levels could influence simple treatments such as scheduling supplements, potentially aiding in long-term muscle and bone growth as well as improving strength and physical performance [3].

### 5.6. Parathyroid Hormone (PTH)

Parathyroid hormone (PTH) is produced and secreted exclusively by the chief cells of the parathyroid gland [80]. Its primary physiological function is to regulate extracellular calcium homeostasis [80]. Elevated levels of PTH (as observed in primary and secondary hyperparathyroidism) have been linked to an increased occurrence of heart failure, hypertension, cardiac arrhythmias, left ventricular hypertrophy, and valvular calcific disease [81]. The study by Meng et al. showed that higher PTH levels were independently associated with an increased risk of heart failure in the general population [82].

PTH has also been studied in the Fontan circulation, primarily in relation to the musculoskeletal system, similarly to vitamin D.

In the above-mentioned study from 2015 by Avitabile et al., the authors studied bone density and structure in 43 patients with the Fontan circulation [73]. Regarding PTH, the study showed that 18 of the enrolled patients had PTH levels above 53 pg/mL (the upper limit of the reference range at the authors’ center). Bone and muscle deficits were not associated with PTH levels.

Sharma et al. studied the prevalence of chronic kidney disease in 68 patients with Fontan circulation (median age 13 (IQR 9.0–17.3 years) and 70 controls [83]. Regarding PTH, the authors found that the median PTH level was significantly higher in Fontan patients compared to healthy controls [59.4 (IQR 43.0–83.1) pg/mL vs. 23.4 (IQR 16.7–30.0) pg/mL, *p* ≤ 0.001]. The authors concluded that the increased prevalence of hyperparathyroidism (and proteinuria) may be indicators of abnormal kidney function in the Fontan population [83].

In the abstract published in the *Circulation* journal by Mancilla et al. (mentioned above), the authors performed a cross-sectional evaluation of 210 Fontan patients [75]. In patients without PLE, the mean level of PTH was 55.5 ± 35.4 pg/mL (reference range: 9–52 pg/mL). In all patients, 47% of them had above-normal PTH levels. Interestingly, PTH values were higher in patients with PLE (PTH > 52 pg/mL in 84% of PLE patients).

Holler et al. studied 28 Fontan patients screened for vitamin D deficiency, finding significantly higher PTH levels in those with protein-losing enteropathy (PLE) (86.4 ± 33.3 ng/L vs. 43.2 ± 22.4 ng/L, respectively; *p* = 0.03) and more frequent hyperparathyroidism defined as circulating PTH levels of >72 ng/L (80% vs. 6.3%, respectively; *p* = 0.001) [74]. PTH levels correlated with relative neutrophil count (r = 0.631, *p* = 0.002) and neutrophil-to-lymphocyte ratio (r = 0.484, *p* = 0.026), suggesting a link between PTH and systemic inflammation in Fontan patients.

The study by D’Ambrosio et al. previously mentioned measured bone mineral density in 28 Fontan patients [77]. In a post hoc correlation analysis, the authors found significant inverse correlations between PTH and corrected calcium (r = −0.46, *p* = 0.02) and 25-OH vitamin D (r = −0.53; *p* = 0.01). Positive correlation was found between PTH and NT-proBNP level (r = 0.40, *p* = 0.05) and aldosterone (r = 0.65, *p* = 0.01). The authors hypothesized that PTH being elevated and negatively correlated with vitamin D levels was highly suggestive of secondary hyperparathyroidism associated with subclinical vitamin D deficiency [77].

All in all, there are no data on the usefulness of parathyroid hormone as a diagnostic/prognostic biomarker in the Fontan circulation. According to the American Heart Association’s scientific statement, the available data suggest that secondary hyperparathyroidism is frequently observed in children with Fontan circulation [3]. This condition may be linked to hypothesized changes in calcium metabolism due to altered renal perfusion or inadequate absorption in the gut. Notably, secondary hyperparathyroidism can contribute to bone demineralization and impaired growth in Fontan population [3]. Interestingly, the authors of the Scientific Statement note that there is evidence for PTH as a biomarker in heart failure and that consequently, there is a potential for PTH to be used as a prognostic marker in the Fontan circulation [3]. However, there are currently no data to support this notion.

### 5.7. von Willebrand Factor (vWF)

The von Willebrand factor (vWF) is a multimeric glycoprotein present in blood plasma, the subendothelial matrix, and in storage granules in endothelial cells and platelets [84]. The protein is primarily recognized for its role in the hemostatic process, facilitating platelet adhesion and aggregation at sites of vascular injury, and transporting coagulation factor VIII (FVIII) in the bloodstream [84]. As the stored vWF is rapidly released at moments of endothelial cell damage, it is considered as a promised biomarker for endothelial dysfunction [85]. According to some studies high plasma levels of the vWF predict a poor prognosis in patients with heart failure [86,87].

von Willebrand factor levels were also measured in patients with Fontan circulation. In 2007, Binotto et al. aimed to look for evidence of impaired endothelial function and fibrinolysis in Fontan circulation patients [88]. The authors enrolled 23 Fontan patients aged from 7 to 26 years (median 14 years). The study showed that Fontan patients had increased levels of von Willebrand factor in comparison to the control group (*p* = 0.003). The authors noted that patients with Fontan circulation may have endothelial dysfunction [88].

In 2014, Tomkiewicz-Pajak et al. performed a comprehensive analysis of blood coagulation, fibrinolysis, and platelet activation in 48 adult Fontan patients [89]. The study showed that the vWF level was 23% higher in Fontan patients than in the control group (150 ± 28% vs. 122 ± 20%, respectively; *p* < 0.001). The authors suggested that increased plasma vWF levels was evidence for endothelial injury in Fontan patients [89]. In 2015, authors from the same center studied platelet function and responsiveness to aspirin in 34 Fontan patients (and 32 age- and sex-matched healthy controls) [90]. In that study plasma levels of the vWF were significantly higher in Fontan patients than in the control group (*p* < 0.001).

In 2020, Ohuchi et al. studied plasma levels of von Willebrand factor antigen (vWF:Ag) in 382 ACHD patients, including 172 with Fontan circulation [91]. Fontan patients had significantly higher vWF:Ag levels; Fontan circulation was also independently associated with high log-transformed vWF:Ag (*p* < 0.0001). In all included ACHD patients, elevated vWF:Ag predicted all-cause mortality (HR 1.63 per 0.1, 95% CI 1.40–1.96, *p* < 0.0001), patients with high vWF:Ag (≥165%) had a substantially higher risk of all-cause mortality (HR 56.4, 95% CI 11.4–1020, *p* < 0.0001). The authors concluded that high vWF:Ag strongly predicts all-cause mortality in ACHD, possibly reflecting right-sided HF severity and liver dysfunction [91].

In the previously mentioned study by van den Bosch et al., the authors included 133 patients with Fontan circulation and aimed to determine the prognostic value of several blood biomarkers, including von Willebrand factor [37]. The study showed that the event-free survival was better in patients with lower levels of the vWF (*p* = 0.008). Furthermore, in a univariable Cox regression model, a higher vWF were associated with severe events during follow-up. The authors noted that none of the observed events were thromboembolic [37]. Meinel et al. studied 34 Fontan patients (aged 5–38 years) to assess the relationship between cholestasis and selected hemostatic factors [92]. Elevated vWF:Ag levels were observed in both cholestatic and non-cholestatic patients without significant differences (*p* = 0.701). However, vWF:Ag levels were significantly lower in patients on acetylsalicylic acid (ASA) compared to those not on ASA (*p* = 0.0436). Additionally, a pathologically low vWF collagen binding capacity/vWF antigen (_V_WF:CB/_V_WF:Ag) ratio, indicating acquired von Willebrand syndrome, was found in 29.4% of patients. The authors concluded that preoperative testing for acquired von Willebrand syndrome should be considered in cholestatic Fontan patients to prevent bleeding events [92].

To sum up, although there are some data on the vWF in the Fontan circulation, no recommendation can be made for routine measurement of this biomarker. Undoubtedly, the role of the vWF should be studied in the context of endothelial pathophysiology and coagulation disorders. However, there is also potential for the prognostic use of this biomarker; therefore, further studies are needed.

## 6. Review of Available Evidence on the Most Promising New Blood Biomarkers in the Fontan Circulation

### 6.1. Carbohydrate Antigen 125 (CA125)

Carbohydrate antigen 125 [CA125, also called cancer antigen 125 or mucin 16] is a complex glycoprotein encoded by the MUC16 gene in humans [93]. This antigen is normally expressed in tissues derived from coelomic epithelia, such as the ovary, peritoneum, fallopian tube, pericardium, pleura, kidney, stomach, and colon [94]. CA125 measurement is primarily used in the management of ovarian cancer—in screening, treatment and follow-up [94]. Recently, however, CA125 has emerged as a reliable marker of congestion and inflammation in patients with heart failure [93]. It has been suggested that CA125 measurement has the potential to both monitor and guide HF treatment after a decompensated HF event [93].

Considering that CA125 identifies patients with heart failure and congestive patterns [95] and that Fontan circulation complications are associated with systemic venous congestion, Fuentes et al. hypothesized that CA125 measurement may be particularly useful in Fontan patients [52]. The authors prospectively enrolled 66 patients with Fontan circulation (mean age 27.4 ± 7.8 years). The median CA125 level was 15.4 (IQR 8.1–32.7) IU/mL. CA125 values were significantly higher in patients with the composite event [30.1 (IQR 21.1–57.4) IU/mL vs. 12.6 (7.9–18.7) IU/mL, respectively; *p* = 0.001]. In the multivariate analysis, logarithmically transformed CA125 (LnCA125) was significantly associated with the occurrence of the composite event [OR = 4.7 (95%CI 1.7–12.8); *p* = 0.002]. The cut-off points with the optimal sensitivity and specificity for LnCA125 were also calculated with a result of ≥3 (which corresponded to CA125 level of ≥20 U/mL). The authors concluded that patients with CA125 levels of ≥20 U/mL [along with RDW ≥ 14.5% and a Fibrosis-4 score (FIB4) ≥0.75] had a very high probability of Fontan circulation failure [52].

All in all, CA125 seems to be a promising biomarker for assessing the state of the Fontan circulation. Certainly, a clear advantage of CA125 measurement is the widespread availability of this biomarker measurement in most clinical laboratories. Future studies may support the routine use of CA125 in Fontan patients.

### 6.2. Lipoproteins

Lipoproteins in plasma transport lipids to tissues for energy utilization, steroid hormone production, lipid deposition, and bile acid formation [96]. There are six major lipoproteins in blood: chylomicrons, very low-density lipoprotein (VLDL), intermediate-density lipoprotein (IDL), low-density lipoprotein (LDL); lipoprotein(a) [Lp(a)], and HDL (high-density lipoprotein) [96]. Although hypercholesterolemia is a serious epidemiologic problem in the general population, hypocholesterolemia (which can be defined as lipoprotein levels below the fifth percentile of the general population adjusted for age, sex, and race [97]) is frequently observed in the Fontan circulation [98,99,100]. Abnormal lipid levels in Fontan patients have been studied in the context of liver disease. Interestingly, evidence suggests that in chronic liver diseases, hypocholesterolemia indicates more severe liver dysfunction, and HDL levels may be the most reliable indicator of the extent of liver disease and its prognosis [100,101,102].

In 2012, Whiteside et al. measured lipoprotein levels in 88 patients with Fontan circulation [median age 14.8 (IQR 10.6–20.0) years] [98]. The study showed that Fontan patients had significantly lower serum total cholesterol, LDL-C, HDL-C and non-HDL-C levels than age- and sex-matched controls. In 2016, authors from the same center studied cholesterol metabolism in 21 patients with Fontan circulation [99]. The study showed that Fontan patients were hypocholesterolemic, with features of increased cholesterol absorption and decreased cholesterol synthesis. The authors noted that cholesterol absorption efficiency was a regulated process; therefore, that finding suggested an up-regulation of cholesterol absorption as a result of decreased cholesterol production [99]. Since cholesterol is synthesized in the liver, hypocholesterolemia may be caused by liver dysfunction from Fontan-associated liver disease (FALD) [100].

In 2021, Lubert et al. hypothesized that reduced cholesterol levels in Fontan patients would correlate with a higher risk of negative cardiac events in these patients [100]. The authors included 164 adult patients with Fontan circulation [median age 30.3 (IQR 22.8–34.4) years] and 81 healthy controls. The study showed that in comparison to the control group, the Fontan patients had a 19% lower LDL-C (82.5 ± 25.4 mg/dL vs. 102.0 ± 34.7 mg/dL, respectively; *p* < 0.0001), 33% lower HDL-C (42.8 ± 12.2 mg/dL vs. 64.1 ± 16.9 mg/dL, respectively; *p* < 0.0001) and 16% lower non-HDL-C (106.1 ± 32.6 mg/dL vs. 126.7 ± 42.5 mg/dL, respectively; *p* < 0.0001). In the univariate survival analysis, lower HDL-C levels were associated with a greater risk of the combined outcome of nonelective hospitalization or death [unadjusted HR per decrease of 10 mg/dL: 1.30; (95%CI, 1.01–1.67); *p* = 0.045]. In a multivariable-adjusted model, HDL-C levels remained associated with a greater hazard for the combined outcome [HR per decrease of 10 mg/dL: 1.37; (95%CI, 1.04–1.81); *p* = 0.0264]. The authors concluded that additional research was required to identify the reasons behind the reduced lipid levels and to assess whether HDL-C could serve as an indicator of more advanced liver disease in patients with Fontan circulation [100].

Finally, in the most recent paper from 2024, Lu et al. found that total cholesterol levels decreased with the increasing severity of liver fibrosis in Fontan patients (Spearman’s alpha: −0.383, *p* = 0.009) [103]. 

Overall, HDL measurement shows potential for monitoring liver function in Fontan patients, though further studies are necessary to provide additional evidence.

### 6.3. Hepatocyte Growth Factor (HGF)

Hepatocyte growth factor (HGF) is a mesenchymal cytokine important to the development of many epithelial and endothelial cells [104]. HGF is released in response to endothelial injury, and there is evidence that elevated levels of HGF are an independent predictor of coronary heart disease (CHD), stroke, progression of atherosclerosis, and also heart failure [104].

HGF was first measured in the Fontan circulation by Mori et al. in 2007 [105]. The authors studied the development of aortopulmonary collaterals in 30 Fontan patients (as well as 29 patients with cyanotic heart disease in the second group and a third 26-patient control group). Regarding HGF, the study showed that serum levels of this biomarker were similar between the three groups.

In 2011, Kim et al. studied the relationship between HGF and protein-losing enteropathy (PLE) after a Fontan operation [106]. The authors enrolled 10 Fontan patients with PLE (study group) and two control groups: one with 20 Fontan patients without PLE and one with 10 patients with nephrotic syndrome. The study showed that the serum HGF level was significantly higher in the PLE patients than in the no-PLE group (0.61 ± 0.27 ng/mL vs. 0.41 ± 0.12 ng/mL, respectively; *p* = 0.024) and nephrotic group (0.61 ± 0.27 ng/mL vs. 0.26 ± 0.12 ng/mL, respectively; *p* = 0.002). The authors concluded that HGF may play a role in the development of PLE after a Fontan operation [106].

Finally, in 2020, Kojima et al. published a study in the *ESC Heart Failure journal*, which aimed to assess the usefulness of the HGF in 34 patients with Fontan circulation (mean age 59.3 ± 7.9 months) [107]. Patients were divided into groups based on HGF levels: normal (<0.4 ng/mL; *n* = 20) and elevated (≥0.4 ng/mL; *n* = 14). The elevated HGF group had significantly higher central venous pressure [CVP] (13.4 ± 0.7 vs. 9.7 ± 0.4 mmHg, respectively; *p* < 0.0001), and HGF correlated positively with CVP (r = 0.33, *p* = 0.0004). In a multivariate analysis, HGF independently predicted both elevated CVP (β-coefficient = 21.2; Standard Error = 5.5; *p* = 0.0005) and decreased arterial oxygen saturation [SvO2] (β-coefficient = −92.9; Standard Error = 12.4; *p* < 0.0001). Interestingly, the receiver-operating characteristic (ROC) curve analysis indicated that HGF > 0.405 ng/mL predicted the need for catheterization with 75% sensitivity and 83.3% specificity. The authors concluded that HGF was an independent predictor of Fontan failure and an indicator for additional catheter intervention after Fontan surgery [107].

Overall, there is some potential for HGF measurement in the Fontan circulation, but additional studies are definitely needed.

## 7. Review of Available Evidence for Other Blood Biomarkers Studied in the Fontan Circulation

### 7.1. Troponins

Troponins are structural proteins in the troponin complex of cardiac muscle, released into the blood upon myocardial injury. Troponins T and I, which are primarily found in the myocardium, are referred to as cardiac troponins (cTnT and cTnI, respectively). High-sensitivity cardiac troponin (hs-cTn) assays are the gold standard for the evaluation of suspected acute myocardial injury [108,109]. Although the main clinical use of hs-cTn measurement is the diagnosis of ischemia, there is also evidence of its prognostic value, not only in coronary artery disease but also in heart failure or valvular heart disease [109]. High-sensitivity troponins have also been studied in the ACHD population. According to the systematic review by Willinger et al., across all included studies, elevated hs-cTn was found to be an independent predictor of survival and heart failure in stable ACHD [110].

There are some data on troponins in the Fontan population. Interestingly, several studies showed that troponins levels were unexpectedly low in Fontan patients [34,39,110,111,112,113]. On the other hand, there were studies in which troponins levels were higher in Fontan patients [59], elevated troponins (>14 ng/L) identified patients at the highest risk of cardiovascular events [34] and were linked with ventricular dilation and dysfunction [38].

The 2021 systematic review published by Willinger et al. [110] emphasized that patients with Fontan circulation, in contrast to other ACHD subtypes, had surprisingly low hs-TnT levels [34,111], while NT-proBNP and GDF-15 values were comparably high [34]. One potential explanation is that the Fontan circulation leads to relatively underloaded ventricle, which may result in less myocardial damage. However, this mechanism is not fully understood [110].

In conclusion, there are few data suggesting that elevated hs-cTn may identify Fontan patients at higher risk of cardiovascular events. Further studies are needed to provide additional evidence.

### 7.2. ST2 Protein

The ST2 (suppression of tumorigenicity 2) gene constitutes a part of the wider interleukin 1 (IL-1) gene cluster [114]. There are two important products of this gene: a soluble form (sST2) and a membrane receptor member of the IL-1 receptor family (ST2L). Interleukin-33 (IL-33) is a functional ligand of ST2L. It is thought that sST2 acts as a decoy receptor and consequently reduces the cardioprotective effects of IL-33 [114]. The measurement of plasma sST2 was studied as a possible biomarker in various cardiac diseases, including HF [114,115]. sST2 values predict reverse remodeling and clinical outcome in patients with chronic HF on guideline-recommended treatment [116]. sST2 measurement is considered a valuable tool for risk stratification in chronic HF, either alone or together with natriuretic peptides and troponins [116].

There were some studies in which sST2 was measured in patients with Fontan circulation. Several articles showed elevated levels sST2 in Fontan patients [38,117,118]. In the study by Laqqan et al., sST2 levels were among the most important predictors of acute heart/Fontan failure [area under curve (AUC) 0.742, 95% CI 0.626–0.858; *p* = 0.004)]. The optimal cut-off for predicting failure was 31.1 ng/mL (sensitivity = 84.6%, specificity = 58.3%) [117]. In the study by van den Bosch et al., higher levels of sST2 were associated with severe events during follow-up [37]. Perrone et al. reported a significant reduction in sST2 levels after a 4-week exercise program in 12 Fontan patients [39].

In conclusion, there are few data on sST2 measurements in Fontan patients, and further studies are needed to provide additional evidence.

### 7.3. Growth Differentiation Factor 15 (GDF-15)

Growth differentiation factor 15 (GDF-15) is a member of the transforming growth factor-β (TGF-β) cytokine superfamily. Under normal conditions GDF-15 is weakly expressed in human tissues; however, it is strongly upregulated in response to hypoxic, mechanical, oxidative, or inflammatory stress [119,120]. GDF-15 has been investigated as a predictive biomarker in various medical conditions, such as ischemic heart disease, heart failure (HF), atrial fibrillation, diabetes mellitus, and cancer [120]. According to the 2022 meta-analysis performed using data pooled from eight trials including 53,486 patients, GDF-15 consistently added prognostic information for cardiovascular death and hospitalization for heart failure across the spectrum of atherosclerotic cardiovascular disease [120].

The usefulness of GDF-15 as a biomarker was also studied in patients with Fontan circulation. Raedle-Hurst et al. (2010) reported significantly higher GDF-15 levels in Fontan patients with an ejection fraction (EF) <50% compared to those with EF ≥ 50% (*n* = 38), identifying GDF-15 as an independent predictor of impaired ventricular function (*p* = 0.006), with a 613 pg/mL cutoff offering 90% sensitivity and 85.7% specificity for predicting EF < 50% [121]. Saraf et al. found elevated GDF-15 in 44 Fontan patients, noting a positive correlation with atriopulmonary Fontan duration (r = 0.55, *p* = 0.01), suggesting it may result from progressive organ fibrosis [59]. Meyer et al. (2020) observed that Fontan patients with GDF-15 levels in the upper quartile had a higher risk of hospitalization or death [HR 2.76, 95% CI 1.27–6.00, *p* = 0.011], with a similar risk increase in patients with GDF-15 above 70 pg/mL after two years [HR 2.69, 95% CI 1.03–6.99, *p* = 0.043] [122]. Van den Bosch et al. associated low GDF-15 levels with better event-free survival in 133 Fontan patients [37], while Perrone et al. found GDF-15 increased after a 4-week exercise program, likely due to physiological muscle stress [39].

To sum up, although we have some evidence of the usefulness of GDF-15, further studies are definitely needed before introducing its measurement into clinical practice.

### 7.4. Galectin-3

Galectin-3 belongs to the galectin family, a crucial group of β-galactoside-binding lectins that play a significant role in modulating “cell–cell” and “cell–matrix” interactions [123,124]. Galectins are important regulators of inflammatory and immune system responses [123]. Multiple studies have shown the increased expression of galectin-3 in hypertrophied hearts, as well as its ability to promote macrophage migration, fibroblast proliferation, and the progression of fibrosis [125]. An increased concentration of galectin-3 was found in patients with chronic HF, regardless of etiology and HF typology [126]. It is considered to be a novel prognostic biomarker with high predictive value for cardiovascular mortality and re-hospitalization in HF patients [124].

In 2016, given the extensive fibrosis seen in Fontan patients, Opotowsky et al. hypothesized that galectin-3 would be elevated in that population and associated with adverse outcomes [127]. In 70 adult Fontan patients, plasma galectin-3 levels were significantly higher than in 21 matched controls [11.85 (IQR 9.9–15.0) ng/mL vs. 9.4 (IQR 8.2–10.8) ng/mL; *p* < 0.001]. Patients with galectin-3 > 14.3 ng/mL faced a higher risk of hospitalization or death [HR 6.0, 95% CI 2.1–16.8; *p* < 0.001], even after adjusting for covariates. The authors concluded that elevated galectin-3 levels in Fontan patients were linked to increased adverse outcomes [127]. On the other hand, in the above-mentioned study by van den Bosch et al., involving 133 Fontan patients, galectin-3 levels showed no association with clinical events or cardiac function parameters during follow-up [37].

Overall, we have very few data on galectin-3 in the Fontan circulation. Further studies are needed to assess the utility of this potential biomarker.

### 7.5. Adrenomedullin (ADM)

Adrenomedullin (ADM) is a peptide hormone, which was discovered in 1993 by Kitamura et al. [128]. The most recognized function of ADM is vasodilatation in both vascular capacitance and resistance vessels [129]. ADM also seems to play an important role in the preservation of endothelial integrity [129]. Elevated levels of ADM have been linked to unfavorable clinical outcomes in various studies involving heart failure patients [129]. The majority of studies measure a stable part of the ADM precursor peptide: mid-regional pro-ADM (MR-proADM) [129,130].

The first adrenomedullin (ADM) measurements in Fontan patients were published by Hiramatsu et al. (1999), showing lower ADM levels in the early post-operative setting after Fontan completion [131]. Watanabe et al. (2007) found significantly elevated venous ADM levels in 29 Fontan patients relative to age-matched controls [132]. Kaiser et al. studied MR-proADM levels in 53 Fontan patients (mean age 13.1 ± 7.2 years) and found significantly higher levels in those with Fontan failure (0.668 nmol/L vs. 0.357 nmol/L, respectively; *p* =  0.001) [130]. The receiver operating characteristic (ROC) curve analysis showed that MR-proADM could predict Fontan failure (AUC 0.985, *p* =  0.001) with an optimal cutoff of 0.520 nmol/L, yielding 100% sensitivity and 93.9% specificity. The authors suggested that serial MR-proADM measurements could help identify patients at risk for Fontan failure [130]. Interestingly, Hauser et al. (2021) observed that MR-proADM levels could be affected by the ingestion of a meal and recommended measuring the biomarker under fasting conditions for consistent assessment [66].

All in all, although the potential of MR-proADM measurement for identifying high-risk patients is interesting, we definitely need new studies with larger samples to provide additional evidence.

### 7.6. Endothelin-1 (ET-1)

Endothelin-1 (ET-1) is synthesized and released continuously from endothelial cells, including pulmonary epithelial cells [133,134]. ET-1 affects blood pressure via vasoconstriction and plays a crucial role in the control of basal vascular tone [134]. ET-1 plasma levels are elevated in patients with primary pulmonary hypertension (PPH) [135]. Drugs that antagonize the ET-1 system—ET-1 receptor antagonists—are used in patients with PPH [136]. ET-1 may serve as a potential prognostic biomarker for developing pulmonary hypertension in HF with preserved ejection fraction [137].

Pulmonary vascular resistance is the key factor for effective Fontan circulation, as even a small increase in pulmonary vascular resistance can result in significantly reduced cardiac output [138]. As ET-1 levels correlate with pulmonary pressures, this biomarker has been studied in Fontan patients.

Elevated ET-1 levels in Fontan patients were observed in several studies [66,90,131,138,139,140]; in one study, only in Fontan patients with reduced cardiac function [141]. In 2011, Kolcz et al. showed that endothelin-1 levels correlated significantly with the respiratory equivalent of carbon dioxide at peak exercise (VE/VCO_2peak_) [r = 0.84; *p* = 0.008] in 76 patients after the total cavopulmonary connection (TCPC) type of Fontan operation [29].

In summary, there is a paucity of data on ET-1 in the Fontan circulation. Further studies are needed to evaluate the usefulness of ET-1 as a biomarker. Nevertheless, it is important to note that ET-1 receptor antagonists (such as bosentan) are used in Fontan patients with increased pulmonary pressure/resistance. These drugs are even part of the ESC ACHD guidelines recommendations (class 2b, level C) [1].

### 7.7. Components of the Renin–Angiotensin–Aldosterone System (RAAS)

The renin–angiotensin–aldosterone system (RAAS) plays an important role in controlling blood volume, maintaining electrolyte balance, and regulating systemic vascular resistance. Its main components are renin, angiotensin I and II, and aldosterone. The disruption of the RAAS in humans results in damage and fibrosis in various organs, including the kidneys, heart, and vascular walls [142]. Serum levels of RAAS components can be measured and used as biomarkers. For example, plasma renin is considered a risk factor for cardiovascular events in the hypertensive population and in high-risk patients [143].

Elevated levels of RAAS components in Fontan patients were reported in several studies [20,59,144]. Two studies found an association between overactivation of the RASS and pleural effusions after a Fontan operation [145,146].

Ohuchi et al. found that high renin activity in Fontan patients was associated with diuretic use and low arterial pressure, suggesting that the RAAS plays an important role in maintaining perfusion pressure in these patients [19]. Inai et al. found a negative correlation between angiotensin II levels and left ventricular ejection fraction, suggesting angiotensin II may indicate ventricular damage from volume overload and/or prior cyanosis in Fontan patients [20]. In 2011, Ohuchi’s et al. study linked hyponatremia in Fontan patients to high renin activity and diuretic use, pointing to hypovolemia-induced RAAS activation [147]. Burchill et al. found that a “high-risk” RAAS genotype in 106 Fontan adults was associated with diastolic dysfunction and higher serum BNP levels [30]. Interestingly, Sugimoto et al. found positive correlations between RAAS components and procollagen type III N-terminal amino peptide (PIIIP) levels, a fibrosis marker, in Fontan patients, suggesting that the RAAS inhibition might help prevent ventricular fibrosis post-Fontan surgery [148].

In conclusion, there are no data on the serum levels of RAAS components in determining the state of the Fontan circulation. Although the RAAS has been studied extensively in recent years, much remains unknown about its physiology in the unique Fontan circulation. Therefore, further studies of the RAAS in the Fontan circulation are needed.

### 7.8. Norepinephrine (NE)

Norepinephrine (NE), also known as noradrenaline, is one of the three endogenous catecholamines (along with epinephrine and dopamine). Catecholamines, which are released from the adrenal medulla and the central and sympathetic nervous systems, act as hormones and neurotransmitters and play an important role in the regulation of the cardiovascular system [149]. In heart failure, the activation of the sympathetic nervous system results in an increased circulating concentration of norepinephrine. Elevated NE levels result in increased myocardial contraction, peripheral vasoconstriction, increased heart rate, and increased energy expenditure [150]. NE has been studied as a biomarker in congestive HF with promising results in early studies [151] followed by negative results in later articles [152].

Norepinephrine has also been studied in the Fontan circulation. Ohuchi et al. studied neurohormone levels in 97 clinically stable Fontan patients and found that norepinephrine in particular (in addition to BNP) differentiated the NYHA II group from the NYHA III+IV group. NE levels were also significantly higher in patients with a single functional ventricle of right ventricular morphology compared to patients with a single functional ventricle of left ventricular morphology [19]. In 2011, the same authors found significantly elevated NE levels in 169 hyponatremic Fontan patients [147]. Ohuchi et al. also found that Fontan patients had significantly higher plasma NE levels than biventricular repair patients, with NE levels being the sole predictor of unscheduled cardiac events [153]. Inai et al. studied 50 Fontan patients and found significantly higher NE levels compared to controls, with NE negatively correlated with left ventricular ejection fraction and positively with pulmonary arterial wedge pressure [20]. A multivariate analysis indicated that NE, alongside ejection fraction, predicted survival and cardiac event-free rates. In Kaplan–Meier event-free survival curves, NE combined with peak oxygen consumption (peak VO_2_) showed a significant predictive value. The authors concluded that their findings suggested that high adrenergic activity and poor exercise capacity contributed to a poor prognosis in Fontan patients [20]. Finally, in a 2015 study, Ohuchi et al. found that serum NE serum norepinephrine (per 100 pg/mL) predicted all-cause mortality [HR = 1.32 (95% Cl 1.15–1.50), *p* = 0.0002] [31].

In conclusion, there is little evidence to support the use of NE as a biomarker in the Fontan circulation. Its measurement is useful in studies assessing the pathophysiology of the autonomic nervous system, but there is almost no evidence that NE levels correlate with clinical status.

### 7.9. Pro-Inflammatory Cytokines (IL-6, TNF-α)

Interleukin 6 (IL-6) is a cytokine with a pleiotropic effects on inflammation, immune response, and hematopoiesis [154]. Elevated IL-6 levels are strongly associated with heart failure, atherosclerosis, acute coronary syndromes, and ischemic strokes [155].

Tumor necrosis factor α (TNF-α) is a multifunctional cytokine protein recognized as an important mediator of cell differentiation, proliferation, and survival [156]. In the cardiovascular system, TNF-α-activated signaling pathways play a role in the development of atherosclerosis, vascular dysfunction, adverse cardiac remodeling following a myocardial infarction, and hypertension [157].

Both IL-6 and TNF-α have also been studied in the Fontan circulation. IL-6 and TNF-α have been found to be elevated in the postoperative period after a Fontan operation [158,159]. These cytokines were also significantly elevated in patients with protein-losing enteropathy (PLE) [160,161].

Miyamoto et al. enrolled 103 ACHD patients of which 53 had the Fontan circulation; the authors found that elevated levels of IL-6 were significantly associated with mortality in a univariate analysis in patients with a systemic ventricle of left ventricular morphology [HR 2.97 (95% CI 1.51–5.84), *p* = 0.001] [32]. Saraf et al. found that Fontan patients (*n* = 44) had significantly elevated levels of IL-6 and TNF-α compared to controls. Interestingly, the authors noted that these pro-inflammatory biomarkers were elevated in patients considered to be clinically stable, suggesting that Fontan patients may experience chronic subclinical inflammation throughout their lives [59].

To sum up, there is little evidence to support the use of interleukin 6/tumor necrosis factor α as a biomarker in the Fontan circulation. However, inflammation undoubtedly plays a role in the pathophysiology of the Fontan circulation. Further studies are needed to assess the burden of inflammatory processes in this unique population.

### 7.10. Uric Acid

Uric acid is the end-product of purine metabolism. While experimental studies have identified uric acid as an antioxidant, it is also well established that uric acid triggers inflammation in vascular endothelial and smooth muscle cells, as well as intracellular oxidative stress, which contributes to endothelial dysfunction [162]. Elevated serum uric acid level is associated with cardiovascular diseases such as coronary artery disease, hypertension, and heart failure and is useful for risk stratification [162]. Numerous large-scale population studies have shown that uric acid is an independent predictor of mortality in both acute and chronic heart failure [163].

Uric acid was also studied in Fontan physiology. Ohuchi et al. examined hyperuricemia (defined as uric acid ≥ 7.0 mg/dL) in 197 pediatric and 102 adult Fontan patients [31]. Overall, 22% of included patients showed hyperuricemia; the prevalence of hyperuricemia was significantly higher in adults (*p* = 0.0003). Uric acid levels were inversely correlated with peak VO_2_ and positively with VE/VCO_2_. Hyperuricemia predicted mortality in the univariate analysis [HR = 3.22 (95% CI 1.22–8.65), *p* = 0.0193] but was not a significant predictor in the multivariate analysis [31]. Opotowsky et al. found elevated uric acid levels in patients with high galectin-3 [127]. In the recent study by Michel et al., the authors investigated serum proteomics in a cohort of 20 adult Fontan patients (and 20 matched controls) [164]. Regarding uric acid, the study showed that Fontan patients had significantly higher levels of this metabolite compared to the control group.

Overall, there is only one study suggesting uric acid as a prognostic biomarker in Fontan patients. Certainly, further studies of this metabolite in the Fontan circulation are needed.

## 8. Conclusions and Future Perspectives

In this article, we comprehensively reviewed all available evidence on serum biomarkers in the Fontan circulation. Each of the biomarkers discussed are summarized in Table 1. Unfortunately, we did not find strong enough data to propose evidence-based recommendations. Certainly, robust longitudinal studies are needed to determine the sensitivity, specificity, and overall predictive value of different biomarkers in monitoring Fontan physiology.

Significantly elevated levels of BNP/NT-proBNP are most likely associated with failure of the Fontan circulation. BNP/NT-proBNP measurement is already recommended by the AHA Scientific Statement [3] and is routinely used in clinical practice. Nevertheless, new large prospective studies are urgently needed to assess the sensitivity, specificity and evidence-based cut-off values for this biomarker. Another promising biomarker is the red blood cell distribution width (RDW), which is automatically calculated in a standard complete blood count. An elevated RDW may not only be an indicator of iron deficiency (which should be confirmed by appropriate tests and treated if confirmed), but also an indicator of worse Fontan circulation. Of course, further studies are also required in this case.

Several additional blood biomarkers may be useful in clinical practice. Cystatin C appears to be a valuable tool for assessing renal function in Fontan patients. Highly sensitivity C-reactive protein is not only routinely measured to monitor inflammatory processes but also has potential as a prognostic predictor. Vitamin D and parathyroid hormone should be measured in Fontan patients in the context of musculoskeletal and endocrine health. Finally, von Willebrand factor should be measured if there is a hematologic indication. Interestingly, Meinel et al. also suggested to consider preoperative testing for acquired von Willebrand syndrome in cholestatic Fontan patients to prevent bleeding events [92].

Nearly all studies of biomarkers in the Fontan circulation have several significant limitations [8]. First, patient numbers are generally limited due to the rarity of the condition. As a result, it has been difficult to study the association of biomarkers with major outcomes such as death. Second, serum concentrations of biomarkers are typically measured only once per patient. In addition, differences in serum concentrations between at-risk and standard Fontan patients are typically small and outcomes are limited in number. Another problem is the heterogeneity of Fontan patients [9]. Beginning with differences in the type of Fontan operation and systemic ventricle (which may be of left ventricular morphology, right ventricular morphology, or undetermined) and continuing with issues such as coexisting conditions and Fontan complications. Studies of biomarkers in the Fontan circulation must take these variables into account.

The development of a clinical score/risk prediction algorithm for Fontan patients seems to be a promising concept for future studies. A model using the value of blood biomarker(s) and other clinical parameters could be an extremely valuable tool for daily clinical use. Ideally, only routinely measured parameters would be used in case of serum biomarkers, tests such as NT-proBNP, RDW, or troponin T.

Evidence-based recommendations for the Fontan circulation are scarce. At the same time, the number of Fontan patients increases continuously. Therefore, there is a clear need for continuing further, wide-reaching research in this unique population. The authors of this article agree with Schumacher et al. [8] that it is time for more prospective, longitudinal, and multicenter studies to address the growing problem of an aging Fontan population.

## Figures and Tables

**Table 1:** Summary of the reviewed biomarkers.

Biomarker/Biomarkers Group	Correspondence to Pathophysiological Mechanism	Available Cut-Off Value(s) [for Serum/Plasma] *	Studies in the Fontan Circulation	Clinical Use in Fontan Patients
Natriuretic peptides	Mechanical stress (primarily), systemic ischemia and hypoxia, neurohumoral factors [17]	ESC HF guidelines: BNP < 35 pg/mL, NT-proBNP < 125 pg/mL make a diagnosis of chronic HF unlikely [18] NT-proBNP > 100 pg/mL as a sensitive marker for identification of Fontan patients with significant ventricular dilation or dysfunction (Ghelani et al. [38]) NT-proBNP levels ≥ 300 pg/mL associated with higher Fontan pressure and PAWP during exercise (Miranda et al. [41])	[19,20,21,22,23,24,25,26,27,28,29,30,31,32,33,34,35,36,37,38,39,40,41,42]	AHA Scientific Statement: the authors recommend measuring serum BNP/NT-proBNP levels once in childhood, every 1–3 years in adolescents, and every 1–2 years in adults [3] Age-adjusted NT-proBNP has the potential for a direct quantitative comparison of ventricular stress in pediatric patients [42] Significantly elevated levels of BNP/NT-proBNP are most likely associated with the failure of the Fontan circulation
Red blood cell distribution width (RDW)	Disrupted erythropoiesis [165]	General population: 11–15% [166] Patients with RDW levels ≥ 14.5% have a very high probability of Fontan circulation failure (Fuentes et al. [52])	[50,51,52]	Since the RDW is automatically calculated in a standard complete blood count, clinicians should pay attention to the RDW value An increased RDW could be an indicator of iron deficiency Potential as a prognostic predictor, but more studies are needed
Cystatin C	Renal insufficiency (estimation of the glomerular filtration rate) [53]	General population: 0.61–1.01 mg/L [167]	[56,57,58,59,60]	Cystatin C appears to be a valuable tool for assessing renal function in Fontan patients
High-sensitivity C-reactive protein (hs-CRP)	Inflammation [62]	General population: <1 mg/L—desirable level, reflects a low systemic inflammatory status and lower atherosclerotic risk; 1–3 mg/L—moderate vascular risk; >3 mg/L—higher vascular risk; >10 mg/L—transient infectious process or other acute-phase response [168]	[32,64,66,67]	Some potential as a prognostic predictor, but more studies are needed Should be used (mainly normal CRP) to monitor inflammatory processes as in the general population
Vitamin D	Abnormalities in calcium, phosphorus, and bone metabolism [169]	General population [25(OH)D level]: deficiency: <20 ng/mL; insufficiency: 21–29 ng/mL; sufficiency: 30–100 ng/mL [169]	[72,73,74,75,76,77,78,79]	Should be measured in Fontan patients in the context of musculoskeletal health. If found deficient, vitamin D should be supplemented according to recommendations for the general population; control measurements are recommended. Further studies are needed to evaluate the usefulness of vitamin D as a prognostic biomarker
Parathyroid hormone (PTH)	Overproduction of PTH leads to hypercalcemia [81]	General population: depends on the assay used e.g., for 2nd generation Abbott assay: 15.0–68.3 pg/mL; for 3rd generation DiaSorin assay: 5.5–38.4 pg/mL [170]	[73,74,75,77,83]	Should be measured in Fontan patients if it is necessary for endocrinological reasons Further studies are needed to evaluate the usefulness of PTH as a prognostic biomarker
von Willebrand factor	Endothelial dysfunction [85]	General population: reference range for VWF antigen level (VWF:Ag): 50–200 IU/dL [171]	[37,88,89,90,91,92]	Should be measured if there is a hematologic indication Meinel et al. suggested considering preoperative testing for acquired von Willebrand syndrome in cholestatic Fontan patients to prevent bleeding events [92]
CA125	Increased hydrostatic pressures, mechanical stress, and cytokine activation [93]	General population: a cutoff of 35 U/mL (value used mainly in the context of oncological disease) [52] Fontan patients: patients with CA125 levels ≥ 20 U/mL have a very high probability of Fontan circulation failure [52]	[52]	CA125 seems to be a promising biomarker for assessing the state of the Fontan circulation. Future studies may support the routine use of CA125 in Fontan patients.
Lipoproteins	Hypocholesterolemia as a marker of liver dysfunction [98]	Hypocholesterolemia can be defined as lipoprotein levels below the 5th percentile of the general population adjusted for age, sex, and race [97]; for example, in case of HDL-C (25–29 years): <35 mg/dL for men, <39 mg/dL for women [172]	[98,99,100,103]	HDL measurement shows potential for monitoring liver function in Fontan patients, though further studies are necessary to provide additional evidence
Hepatocyte growth factor	Endothelial injury [104]	General population: normal range of 0–0.4 ng/mL [107] Fontan patients: HGF level > 0.405 ng/mL could predict the need for catheterization with a sensitivity of 75.0% and a specificity of 83.3% (Kojima et al.) [107]	[105,106,107]	Further studies are needed to evaluate the usefulness of hepatocyte growth factor as a prognostic biomarker
Troponins	Myocardial injury [109]	Myocardial ischemia: rise and/or fall in cTn above the 99th percentile of healthy individuals (exact numbers depend on assay used) [173]	[34,38,39,59,110,111,112,113]	Limited use, may suggest myocardial injury, but more data are needed
ST2 protein	Extracellular fibrosis and inflammation [174]	United States population: 8.6–49.3 ng/mL for males, 7.2–33.5 ng/mL for females [175] The optimal cutoff of sST2 levels for the prediction of acute heart/Fontan failure: 31.1 ng/mL (Laqqan et al. [117])	[37,38,39,117,118]	Some potential as a prognostic predictor, but more studies are needed
Growth differentiation factor 15 (GDF-15)	Hypoxic, mechanical, oxidative or inflammatory stress [119]	Males (general population): [median (97.5th centile)] at age < 30 years: 537 (1.135) pg/mL; 50–59 years: 931 (2.492) pg/mL [176] Females (general population): [median (97.5th centile)] at age < 30 years: 628 (2.195) pg/mL; at 50–59 years: 881 (2.323) pg/mL [176] Fontan population: 613 pg/mL as the optimal cutoff for the prediction of EF < 50% (Raedle-Hurst et al. [121])	[37,39,59,121,122]	Some potential as a prognostic predictor, but more studies are needed
Galectin-3	Extracellular fibrosis and inflammation [174]	General population: upper reference limit (97.5th percentile) of 26.1 ng/mL [177]	[37,127]	Few data on galectin-3 in the Fontan circulation, more studies are needed
Adrenomedullin (MR-proADM)	Endothelial dysfunction; residual tissue congestion [129]	General population: depends on assay used; for example, MR-proADM (BRAHMS AG, Hennigsdorf, Berlin, Germany) normal reference range 0.33 ± 0.7 nmol/L [178]	[66,130,131,132]	Some potential in identifying high-risk Fontan patients, but more studies are needed
Endothelin-1 (ET-1)	Vasoconstriction, especially in the pulmonary circulation [179]	General population: 0.7–5 pg/mL [180]	[29,66,90,131,138,139,140,141,181]	Further studies are needed to evaluate the usefulness of ET-1 as a biomarker
Components of the RAAS	Myocardial and vascular remodeling: hypertrophy, fibrosis [182]	General population- plasma renin activity: 0.25–5.82 ng/mL/h; angiotensin II: 10–50 ng/L; aldosterone (>18 years, upright position, 8:00–10:00 AM): <28 ng/dL [183]	[19,20,30,59,144,145,146,147,148]	Further studies are needed to evaluate the usefulness of the RAAS components as prognostic biomarkers
Norepinephrine (NE)	Increased myocardial contraction, peripheral vasoconstriction, heart rate, energy expenditure [150]	General population: 54–393 pg/mL [184]	[19,20,31,147,153]	Further studies are needed to evaluate the usefulness of the norepinephrine as a prognostic biomarker
Pro-inflammatory cytokines (IL-6, TNF-α)	Inflammation	General population- IL-6: 0–5.740 pg/mL [185]; TNF-α, upper 95% percentile (Valaperti et al.): 11.22 pg/mL (Luminex Merk assay) [186]	[32,59,158,159,160,161]	Further studies are needed to evaluate the usefulness of pro-inflammatory cytokines as prognostic biomarkers
Uric acid	Inflammation in vascular endothelial and smooth muscle cells, intracellular oxidative stress, endothelial dysfunction [162]	General population: cutoff of 7 mg/dL in males and 6 mg/dL in females for hyperuricemia [187]	[31,127,164]	Further studies are needed to evaluate the usefulness of uric acid as a prognostic biomarker

* When the reference intervals are given, note that reference intervals may vary between laboratories; therefore, clinicians should be aware of the reference range for the test at the laboratory performing the test. Abbreviations: AHA—American Heart Association; BNP—brain natriuretic peptide; CA125—carbohydrate antigen 125 (also called cancer antigen 125); CRP—C-reactive protein; cTn—cardiac troponin; EF—ejection fraction; ESC—European Society of Cardiology; ET-1—endothelin-1; GDF-15—growth differentiation factor 15; HDL-C—high-density lipoprotein-cholesterol; HF—heart failure; HGF—hepatocyte growth factor; hs-CRP—high-sensitivity C-reactive protein; IL-6—interleukin 6; MR-proADM—mid-regional pro-adrenomedullin; NE—norepinephrine; NT-proBNP—N-terminal prohormone of brain natriuretic peptide; PAWP—pulmonary artery wedge pressure; PTH—parathyroid hormone; RAAS—renin–angiotensin–aldosterone system; RDW—red blood cell distribution width; sST2—soluble suppression of tumorigenicity 2; VWF—von Willebrand factor; 25(OH)D—25-hydroxyvitamin D.

## Data Availability

Not applicable.
